# Effects of recombinant adenovirus-mediated expression of IL-2 and IL-12 in human B lymphoma cells on co-cultured PBMC

**DOI:** 10.1186/1479-0556-2-15

**Published:** 2004-10-14

**Authors:** Oliver Ebert, Dorothee Wilbert, Peter Buttgereit, Carsten Ziske, Dimitri Flieger, Ingo GH Schmidt-Wolf

**Affiliations:** 1 Medizinische Klinik und Poliklinik I, Rheinische Friedrich-Wilhelms-Universität, Bonn, Germany; 2 Medizinische Klinik II, Klinikum Aschaffenburg, Germany; 3 Department of Gene and Cell Medicine, Mount Sinai School of Medicine, New York, New York, USA

## Abstract

**Background:**

Modulation of the immune system by genetically modified lymphoma cell vaccines is of potential therapeutic value in the treatment of B cell lymphoma. However, the anti-tumor effect of any single immunogene transfer has so far been limited. Combination treatment of recombinant IL-2 and IL-12 has been reported to be synergistic for inducing anti-tumor responses in solid tumors but the potential of IL-2/IL-12 gene modified B cell lymphoma cells has not been explored yet.

**Methods:**

Using three different human B cell lymphoma cell lines and primary samples from patients with B cell neoplasms, expression levels of the coxsackie B-adenovirus receptor (CAR) and alpha (v) integrins were analyzed by fluorescence-activated cell sorter (FACS). Adenoviral transduction efficiencies were determined by GFP expression analysis and IL-2 and IL-12 cytokine production was quantified by enzyme-linked immunosorbent (ELISA) assays. Proliferative activities of peripheral blood mononuclear cells (PBMC) stimulated with either cytokine derived from supernatants of transduced lymphoma cells were measured by cell proliferation (MTT) assays. An EuTDA cytotoxicity assay was used to compare cytotoxic activities of IL-2 and/or IL-12 stimulated PBMC against unmodified lymphoma cells.

**Results:**

We found that B cell lymphoma cell lines could be transduced with much higher efficiency than primary tumor samples, which appeared to correlate with the expression of CAR. Adenoviral-expressed IL-2 and IL-12 similarly led to dose-dependent increases in proliferation rates of PBMC obtained from healthy donors. IL-2 and/or IL-12 transduced lymphoma cells were co-cultured with PBMC, which were assayed for their cytolytic activity against unmodified lymphoma cells. We found that IL-2 stimulated PBMC elicited a significant anti-tumor effect but not the combined effect of IL-2/IL-12 or IL-12 alone.

**Conclusion:**

This study demonstrates that the generation of recombinant adenovirus modified lymphoma cell vaccines based on lymphoma cell lines expressing IL-2 and IL-12 cytokine genes is technically feasible, induces increases in proliferation rates and cytotoxic activity of co-cultured PBMC, and warrants further development for the treatment of lymphoma patients in the future.

## Background

Lymphoma cells are attractive targets for gene transfer, because these cells are potentially susceptible to immunotherapeutic strategies [1]. Among the various cancer gene therapies using a variety of genes with different gene transfer systems, immunogene therapy focuses on the use of genes for cytokines, chemokines, and co-stimulatory molecules [2]. Using an ex vivo approach of tumor cell transduction, it was shown that many cytokines could modulate tumorigenicity and protect the host from untreated tumor cells [3]. However, the effect of any single immunogene transfer has been limited, especially against low immunogenic tumors [4].

Interleukin-2 (IL-2) and interleukin-12 (IL-12) are cytokines that elicit strong antitumor effects by stimulating immune cells, including T cells and natural killer (NK) cells. Although either cytokine stimulates the proliferation of T cells, the production of interferon-γ (IFN-γ) by NK cells, and ultimately the cytolytic activity, the magnitude, and the spectrum of stimulatory effects by IL-2 and IL-12 are different. Although IL-2 is a stronger stimulator of proliferation and cytolytic activity, IL-12 is a stronger inducer of IFN-γ from NK cells and activated T cells. Although the combination of recombinant IL-2 and IL-12 treatment has been reported to be synergistic for inducing anti-tumor responses, systemic administration of these cytokines causes toxic side effects. Recent reports of intra-tumoral co-injection of adenoviral vectors expressing IL-2 and IL-12 demonstrated the regression of pre-established solid tumors with high frequency [5]. However, the significance of IL-2 and IL-12 immunogene therapy of hematopoietic neoplasms such as B cell lymphoma, has not been addressed yet.

Recently, we described an adenoviral protocol accomplishing highly efficient gene transfer to B-lymphoma cell lines [6]. The use of genes or genetically modified cells for therapeutic benefit may have a significant therapeutic role for patients with B cell lymphomas in the future. Adoptive immunotherapy using donor leukocyte infusion to treat aggressive B cell neoplasms in immunosuppressed patients has shown great promise clinically, and studies of idiotypic vaccination in patients with low grade B cell neoplasms are also underway. Results from in vitro and animal studies continue to suggest that it may become possible to use the immune system for therapeutic benefit, and many current basic research strategies in the gene therapy of B cell lymphoma are based on immune modulation of T cells or tumor cells themselves. Other major approaches to gene therapy for B cell malignancies are the introduction of directly toxic or suicide genes into B cells.

In the present study, we have evaluated the relationship between the amount of cytokine production by the combination IL-2 and IL-12 and the in vitro effective anti-tumor activity. Using three different human B cell lymphoma cell lines and primary samples from patients with B cell neoplasms, we transduced both IL-2 and IL-12 genes by adenoviral vectors, and monitored cytokine production and effects on proliferation and cytolytic activity of co-cultured human peripheral blood mononuclear cells (PBMC).

## Methods

### Cell culture and primary lymphoma cells

The following cell lines were analyzed: Raji (human Burkitt lymphoma cell line; obtained from "Deutsche Sammlung von Mikroorganismen und Zellkulturen" (DSMZ), Braunschweig, Germany), Daudi (human Burkitt lymphoma cell line; obtained from DSMZ), and OCI-Ly8-LAM53 (human follicular lymphoma cell line; obtained from R. Levy, Stanford University, CA). The cell lines were grown in RPMI 1640 with Glutamax (Life Technologies, Berlin, Germany) supplemented with 10% heat-inactivated fetal calf serum (FCS) (PAA, Martinsried, Germany), 50 μg/ml streptomycin, and 50 μg/ml penicillin (PAA), and were kept in a humified incubator with 5% CO_2 _at 37°C. Virus propagation was performed in the Ad5 E1-transformed human embryonic retina cell line 911 [7]. This cell line was grown in Dulbecco's modified Eagle's medium (DMEM) (Life Technologies) supplemented with 10% FCS, 50 μg/ml streptomycin, and 50 μg/ml penicillin.

Non-adherent Ficoll-Hypaque (Seromed, Berlin, Germany) separated human PBMC were obtained from whole blood from healthy donors and maintained in RPMI 1640 with Glutamax (Life Technologies) supplemented with 10% FCS (PAA), 50 μg/ml streptomycin, and 50 μg/ml penicillin. Cytokine-induced killer (CIK) cells were generated as described previously [8,9]. In brief, 100 U/ml recombinant interferon-gamma (Boehringer Mannheim, Germany) was added on day 0. After 24 h of incubation, 50 ng/ml of an antibody against CD3, 100 U/ml interleukin-1 (IL-1), and 300 U/ml interleukin-2 (IL-2) (PromoCell, Heidelberg, Germany) were added. Cells were incubated at 37°C in a humified atmosphere of 5% CO_2 _and subcultured every 3 days in fresh complete medium and IL-2.

Five patients diagnosed with lymphoma were included into this study; four patients with chronic B cell lymphocytic leukemia (B-CLL) and one patient with immunocytoma (IC). After informed consent, peripheral blood was obtained and lymphoma cells were isolated by Ficoll-Hypaque (Seromed) density centrifugation. Cell surface antigens were analyzed for the expression of CD19, integrin avβ3, integrin avβ5, and the coxsackie B-adenovirus receptor (CAR). Primary cultures were maintained in liquid culture in RPMI 1640 with Glutamax (Life Technologies) supplemented with 10% heat-inactivated FCS, 50 μg/ml streptomycin, and 50 μg/ml penicillin at 37°C, 5% CO_2 _and could be maintained in culture for 10–12 days.

### Adenoviral transduction of lymphoma cells

Transduction of lymphoma cells with CsCl-purified adenovirus was carried out in 24-well plates with 5 × 10^5 ^cells in 50 μl of PBS plus 1 mM MgCl_2_/1% HS, at different multiplicities of infection (MOI). After 2 hours of incubation at 37°C, 5% CO_2_, 1 ml of complete culture medium was added to the cells. Because no visible toxic effect was observed in comparison with the controls (only PBS plus 1 mM MgCl_2_/1% HS), it was not necessary to remove the virus. Adenoviral transduction of primary lymphoma cells was considered successful if concurrent CD19 expression with green fluorescent protein (GFP) was observed.

### Adenoviral vector preparation

The recombinant adenoviral Ad.GFP vector (pQB-AdBM5GFP), an E1- and E3-deleted replication-defective adenovirus type 5 under control of the cytomegalovirus (CMV) promoter, was purchased from Quantum Biotechnologies (Montreal, Canada). The adenovirus vector (Ad.IL-2) containing the human IL-2 sequence was kindly provided by Frank L. Graham, McMaster University, Hamilton, Ontario, Canada [10]. The E1/E3-deleted recombinant Ad5 vector expresses human IL-2 under control of the CMV immediate early promoter (HCMV IE) and the simian virus 40poly(A) signals (SV40 An). The Ad.Flexi-12 vector contains cDNA that encodes a single-chain protein, called Flexi-12, which retains all of the biological characteristics of recombinant IL-12 [11]. This E1/E3-deleted recombinant adenovirus type 5 was generated using the AdEasy system [12] and was kindly provided by Robert Anderson, Royal Free Hospital School of Medicine, London, UK. Infection of Ad.Flexi-12 can be tracked using GFP expression analysis which is present as an additional expression cassette in the viral genome.

Production of the adenovirus lots was performed as described previously [7]. Briefly, near confluent 911 cell monolayers in 175-cm^2 ^flasks were infected with ~5 plaque-forming units (PFU)/cell in 2 ml of phosphate-buffered saline (PBS) containing 1% horse sera (HS). After 2 hours incubation at 37° in a humidified atmosphere of 5% CO_2_, the inoculum was replaced by fresh medium (DMEM/2% HS). After 48 h, nearly completely detached 911 cells were harvested and collected in 1 ml PBS/1% HS. Virus was isolated by three cycles of flash-freeze thawing. The lysates were cleared by centrifugation at 3000 rpm for 10 minutes. Viruses were then purified on double cesium chloride gradients and stored in PBS/10% glycerol at -80°C.

Plaque assays were essentially performed as described by Graham and Prevec [13]. Briefly, adenovirus stocks were serially diluted in 2 ml of DMEM (Life Technologies) containing 2% HS and added to nearly confluent 911 cells in 6-well plates. After 2 hours of incubation at 37°C, 5% CO_2_, the medium was replaced by F-15 minimal essential medium (Life Technologies) containing 1% agarose (Sigma, Deisenhofen, Germany), 20 mM N-2-hydroxyethylpiperazine-N'-2-ethanesulfonic acid (pH 7.4), 0.0025% L-Glutamine, 5% yeast extract, 8.4% NaHCO_3_, 50 μg/mL streptomycin, 50 μg/mL penicillin, and 2% HS. The titers of the virus stocks were at least 1 × 10^10 ^PFU/ml.

### IL-2 and IL-12 enzyme-linked immunosorbent assays (ELISA)

IL-2 and IL-12 levels in conditioned medium were determined by an enzyme-linked immunosorbent assay (ELISA) method. The ELISA reagents were purchased from Endogen, (Cambridge, USA). Briefly, a microtiter plate was coated with a monoclonal antibody specific for IL-2 or IL-12. The IL-12 antibody recognizes only the p70 heterodimer and neither of the individual subunits, p35 or p40, or the homodimeric form of p40. The cytokines present in samples are bound by the immobilized antibody. After several washes to remove unbound proteins, an enzyme-linked (horseradish peroxidase) polyclonal antibody was added to the wells which binds IL-2 or IL-12. After washing, the substrate solution was added, and the color which developed was measured using a spectrophotometer at a wavelength of 450 nm. The optical density of the samples was then compared to a standard curve.

### Cell proliferation assays

An MTT (3-(4,5-dimethylthiazol-2yl)-2,5-diphenyl tetrazolium bromide) based colorimetic assay [14] was performed to measure the proliferative activity of PBMC stimulated with cytokines either derived from supernatants of transduced Raji cells or recombinant with or without addition of neutralizing anti-IL-2 or anti-IL-12 antibodies. In brief, 2 × 10^5 ^PBMC were incubated in 96-well flat-bottom plates (Nunc, Denmark) in a final volume of 200 μl per well. After 3 days 20 μl of EZ4U reagent (Biozol, Eching, Germany) was added to each well and results were obtained on a multi-well scanning spectrophotometer at 450 nm.

### Cytotoxicity assays

A EuTDA nonradioactive cytotoxicity assay (Wallac, Turku, Finland) was used to compare the cytotoxic activity of IL-2 and IL-12 stimulated PBMC against unmodified lymphoma cells [15]. This assay is a colorimetric alternative to the ^51^Cr release assay. The procedure is based on loading the target cells with a fluorescence enhancing ligand (BATDA, bis(acteoxymethyl)2,2:6,2-terpyridine-6,6-dicarboxylate). The hydrophobic ligand penetrates the membrane quickly and within the cell the esterbonds are hydrolysed to form a hydrophilic ligand (TDA, 2,2:6,2-terpyridine-6,6-dicarboxylic acid) which no longer passes the membrane. After cytolysis the ligand is released and introduced to the europium solution. The europium and the ligand form a highly fluorescent and stable chelate (EuTDA). The measured signal correlates directly with the amount of lysed cells.

Briefly, 2 × 10^6 ^lymphoma cells were washed and resuspended in 2 ml PBS. 4.5 μl BATDA solution was added and incubated at 37° for 30 min. Then, cells were washed 3 times, resuspended in 100 μl PBS, and incubated in 96-well flat-bottom plates (Nunc) at a density of 10,000 cells/well. 100 μl of effector PBL cells of varying cell concentations were added so that effector to target cell ratio ranged from 5:1 to 20:1. After incubation at 37° for 2 h cells were centrifuged for 5 min at 500 × g and 20 μl of the supernatant was transfered to a new flat-bottom plate. 180 μl of Europium solution was added, and after 15 min incubtion at room temperature the fluorescence was measured in a time-resolved fluorometer (Wallac). The percent specific release was calculated from



### Statistical analysis

Wilcoxon matched-pairs test was used to analyze for statistical significance. A p value < 0.05 was considered significant. Data is presented as the mean ± standard error of the mean (SEM).

## Results

### Transduction efficiencies of lymphoma cells and CAR/integrin expression

**Figure 1:**
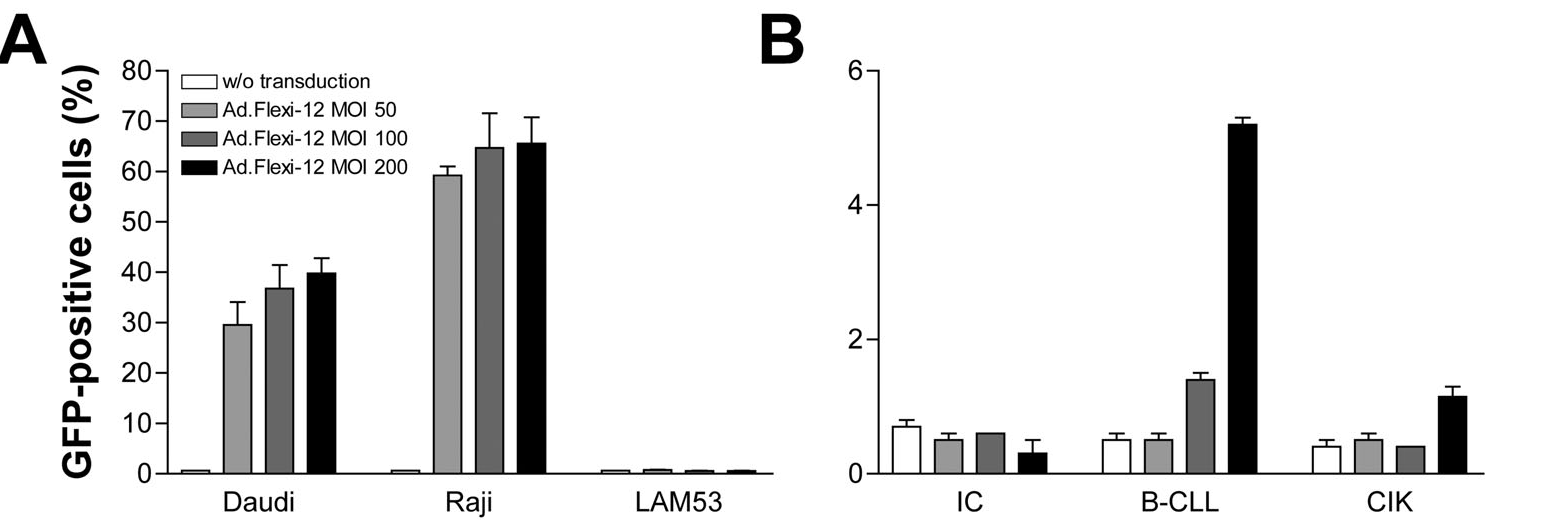
Transduction efficiencies in various human lymphoma cell lines (A), primary human lymphoma cells, and CIK cells (B). All cell types were transduced with Ad.Flexi-12 at various MOI as indicated and analyzed for GFP expression 72 h later by FACS analysis (mean ± SEM; n = 3).

Lymphoma cell lines, primary lymphoma cells, and CIK cells were transduced with Ad.Flexi-12 at various MOI (0, 50, 100, 200) and analyzed 72 h later. Transduction efficiencies were determined by GFP expression analysis using a fluorescence-activated cell sorter (FACS). Additionally, cell surface antigens were analyzed by FACS for the expression of CD19, integrin avβ3, integrin avβ5, and CAR. Adenoviral transduction of primary lymphoma cells was considered successful if concurrent CD19 expression with GFP was observed. It was demonstrated that most B cell lymphoma cell lines could be transduced with much higher efficiency than primary tumor samples or CIK cells. At an MOI of 200, up to 40% of Daudi cells and 70% of Raji cells could be transduced (Fig. 1A). In contrast, primary B-CLL cells were found to be relatively resistant with transduction efficiencies up to 6 %, whereas OCI-Ly8-Lam53 (LAM53) cells, primary IC cells, and CIK cells were completely refractory (Fig. 1B). Transduction efficiency could be correlated with the expression of CAR. High expression of CAR was evident in Raji and Daudi cells, averaging 72% and 86%, respectively. Primary B-CLL cells were found to have moderate CAR expression of 36%. In contrast, there was no CAR expression detectable in LAM53, IC, and CIK cells (Table 1). Expression of integrin receptors, however, was low or absent in all lymphoma cells examined.

**Table 1:** Expression analysis of adenovirus binding (CAR) and internalization receptors (avβ3, avβ5) on various human lymphoma cell lines (Raji, Daudi, OCI-Ly3-LAM53), primary B lymphoma cells (B-CLL, IC), and CIK cells by FACS analysis (mean ± SEM; n = 3; n.d., not detectable).

	**CAR**	**avβ3**	**avβ5**
**Cell lines:**			
Raji	72.5 ± 6.2	1.2 ± 0.1	1.0
Daudi	86.3 ± 1.8	1.1 ± 0.1	1.0
OCI-Ly8-LAM53	3.9 ± 1.5	1.1 ± 0.1	1.0
**Primary cells:**			
B-CLL	36 ± 6.4	0.6	13.6
IC	3.2	n.d.	n.d.
CIK	1.3 ± 0.2	15.0	2.0

### Adenoviral-mediated expression of IL-2 and IL-12 in lymphoma cells in vitro

**Figure 2:**
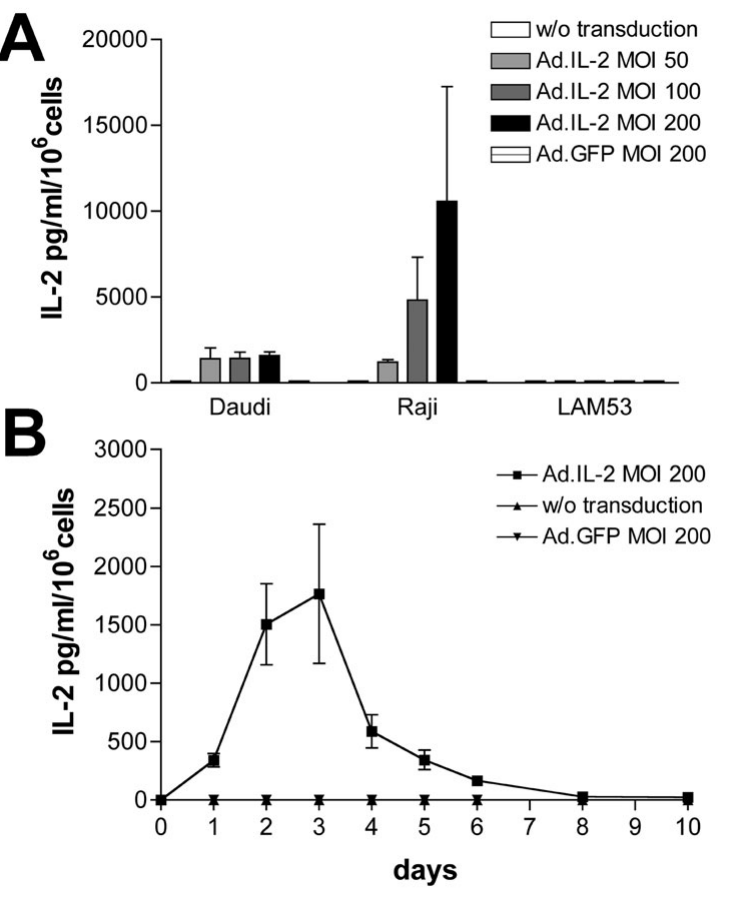
IL-2 gene expression analysis in human lymphoma cell lines by using an ELISA assay. (A) Daudi, Raji and LAM53 cells were infected with Ad.IL-2 or Ad.GFP at various MOI (0, 5, 100, 200). 72 h post-infection, IL-2 produced by Ad.IL-2-transduced Raji and Daudi cells at an MOI of 200 averaged 10.6 ng/ml/10^6 ^cells and 2.7 ng/ml/10^6 ^cells, respectively (mean ± SEM; n = 3). (B) Kinetic analysis of IL-2 production in Raji cells transduced at an MOI of 200 revealed peak secretions between day 2 and 3 and IL-2 was detectable until day 8 post-infection (mean ± SEM; n = 3). All experiments were performed in triplicates.

**Figure 3:**
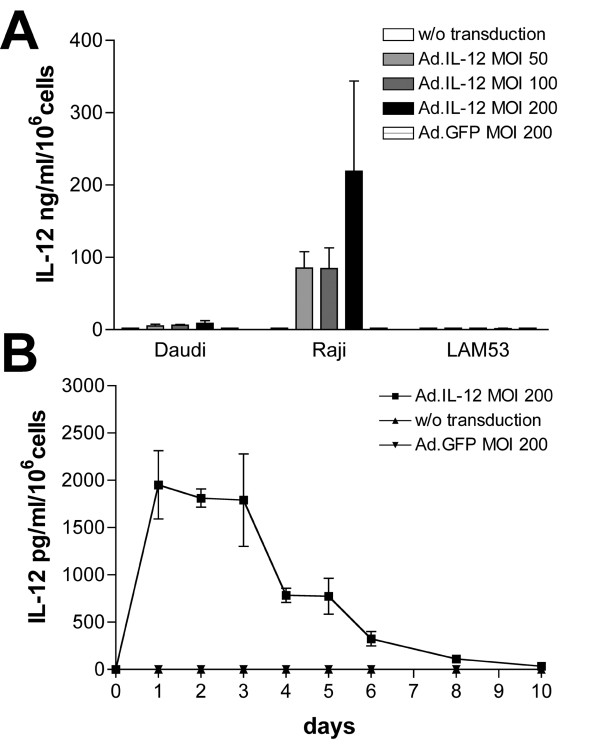
(A) IL-12 gene expression of Ad.Flexi-12 transduced Raji and Daudi cells revealed 219 ng/ml/10^6 ^cells and 15.6 ng/ml/10^6 ^cells at 72 h post-infection, respectively (mean ± SEM; n = 3). No expression was detectable in Ad-Flexi-12 transduced LAM53 cells. (B) Peak expression of IL-12 in transduced Raji cells was evident between day 1 and 3, with IL-12 detectable until day 10 post-infection (mean ± SEM; n = 3). All experiments were performed in triplicates.

Cytokine gene expression was analyzed in lymphoma cell lines using an ELISA assay as described above. Daudi, Raji, and LAM53 cells were infected with Ad.IL-2 or Ad.Flexi-12 at various MOI (0, 50, 100, 200) with Ad.GFP as a control vector. Cytokine production was assayed 72 h post-infection. As shown in Fig. 2A, IL-2 produced by Ad.IL-2-transduced Raji and Daudi cells at an MOI of 200 averaged 10.6 ng/ml/10^6 ^cells and 2.7 ng/ml/10^6 ^cells, respectively. In contrast, there was no IL-2 detectable in Ad.IL-2-transduced LAM53 cells. Kinetic analysis of IL-2 production in Raji cells revealed peak secretions between day 2 and 3. IL-2 was detectable until day 8 post-infection (Fig 2B). Similarly, IL-12 gene expression of Ad.Flexi-12 transduced Raji and Daudi cells revealed 219 ng/ml/10^6 ^cells and 15.6 ng/ml/10^6 ^cells, respectively. No expression was detectable in Ad.Flexi-12-transduced LAM53 cells (Fig. 3A). Peak expression of IL-12 was evident between day 1 and 3, with IL-12 detectable by ELISA until day 10 post-infection (Fig. 3B).

### Increase in proliferation rates of PBMC stimulated with adenoviral-expressed cytokines

**Figure 4:**
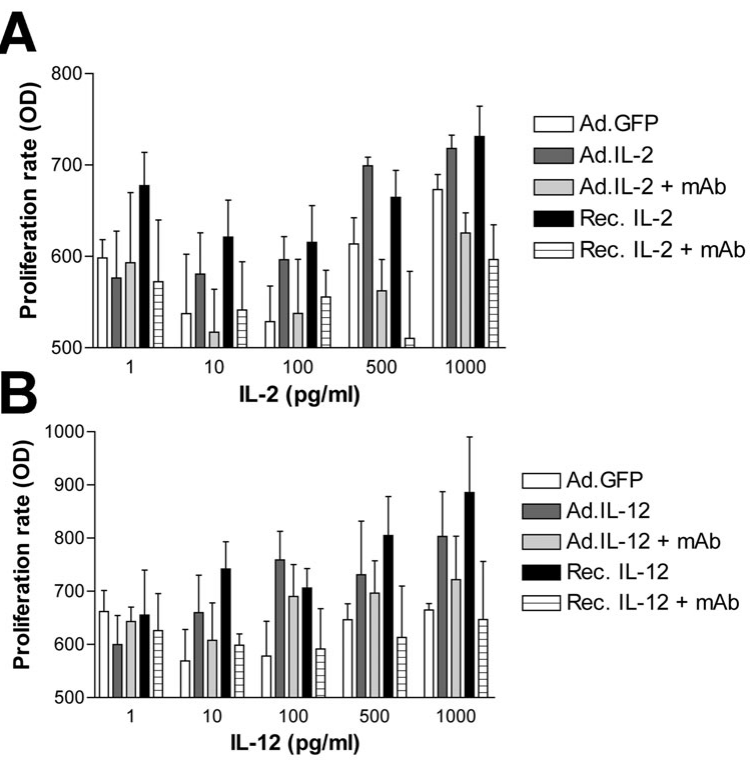
PBMC were incubated with cytokines (1–1000 pg/ml) either derived from supernatants of transduced Raji cells or recombinant with supernatants from Ad.GFP-transduced Raji cells as controls and assayed for their proliferative activity (mean ± SEM; n = 3). Adenoviral-expressed IL-2 (A) and IL-12 (B) led to dose-dependent increases in proliferation rates of PBMC. No significant difference between the effects of either cytokine was found. The proliferation effect could be blocked by addition of a neutralizing antibody against either cytokine. There was no significant difference between the effects of adenoviral-expressed or recombinant cytokines. MTT assays were performed in triplicates.

To determine if adenoviral-expressed cytokines from transduced lymphoma cells would have an impact on the proliferation rates of PBMC from healthy donors, the following experiment was performed. PBMC were freshly isolated and various concentrations of cytokines (1–1000 pg/ml) either derived from the supernatants of transduced lymphoma cells or recombinant were added. Then, an MTT assay to assess the proliferation rate was performed five days later. For blocking experiments, a neutralizing monoclonal antibody against IL-2 or IL-12 was used. Figure 4 shows that addition of adenoviral-expressed IL-2 (Fig. 4A) and IL-12 (Fig. 4B) led to dose-dependent increases in proliferation rates of PBMC. There was no significant difference between the effects of both cytokines. Furthermore, the proliferative effect could be blocked by addition of a neutralizing antibody against either cytokine. Finally, it was demonstrated that there was no significant difference between adenoviral-expressed and recombinant cytokines.

### Cytolytic activity of co-cultured PBMC against unmodified lymphoma cells

**Figure 5:**
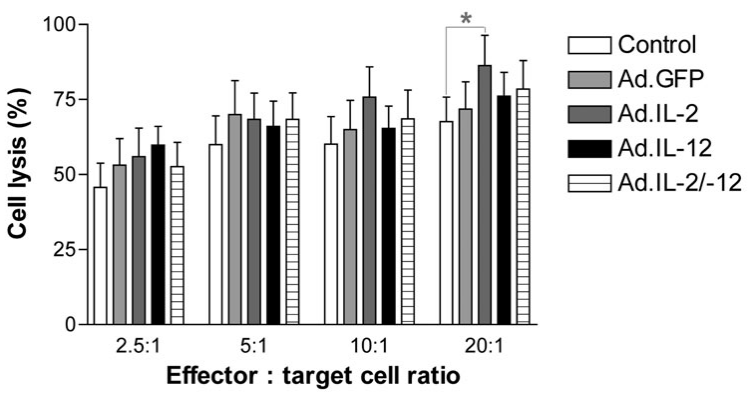
Raji cells were transduced with Ad.IL-2 (MOI 200), Ad.Flexi-12 (MOI 200), or Ad.IL-2 and Ad.Flexi-12 (MOI 100 each) and co-cultured with PBMC for 72 h. Non-transduced (control) and Ad.GFP-transduced Raji cells were used as controls. Stimulated PBMC were harvested and assayed for their cytolytic activity against unmodified lymphoma cells by using an EuTDA non-radioactive cytotoxicity assay. Ad.IL-2-transduced lymphoma cells elicited a significant anti-tumor effect but not the combined effect of IL-2/IL-12 or IL-12 alone (mean ± SEM; n = 5; * p < 0.05).

Raji cells were transduced with Ad.IL-2 (MOI 200), Ad.Flexi-12 (MOI 200), or Ad.IL-2 and Ad.Flexi-12 together (MOI 100 each) and co-cultured with PBMC for 72 h. Non-transduced (control) and Ad.GFP transduced Raji cells were used as controls. Stimulated PBMC were harvested and assayed for their cytolytic activity against unmodified lymphoma cells using a EuTDA nonradioactive cytotoxicity assay. It could be shown that Ad.IL-2 transduced lymphoma cells produced a significant (p < 0.05) anti-tumor effect but not the combined effect of Ad.IL-2/Flexi-12 or Flexi-12 alone (Fig. 5).

## Discussion

The rationale for genetically modified lymphoma cell vaccines is to augment the immunogenicity of poorly immunogenic lymphoma cells, thereby eliciting a systemic immune reponse that is capable of controlling the disseminated disease. Transgene candidates to potentially achieve that goal include genes encoding for cytokines, lymphotactic chemokines, allogeneic MHC molecules, or co-stimulatory molecules [4]. The co-stimulatory molecule CD40 ligand expressed from a recombinant adenoviral vector in autologous chronic lymphocytic leukemia cells has been tested in a recent clinical trial with encouraging results. [16]. Immunotherapy that combines two or more of these immunostimulatory molecules will likely prove more effective than single agents [17]. In this regard, adenoviral-mediated expression of both the IL-2 and IL-12 cytokine genes in several solid tumor models has been found to induce strong and specific anti-tumor responses [5,18]. Interestingly, Wang et al. demonstrated that IL-2 enhances the reponse of NK cells to IL-12 through up-regulation of the IL-12 receptor, signal transducer, and transcription protein STAT4 [19]. Therefore, we were interested in evaluating the potential of IL-2 and IL-12 transduced lymphoma cells for their ability to stimulate and activate immunologic effector cells.

Lymphoma cells are relatively resistant to transduction with most currently available vector systems [20,21]. This problem may be overcome ex vivo by using Epstein-Barr virus vectors [22], adeno-associated virus vectors [23], or modified adenoviral vectors [24,25]. Recently, we described a transduction method accomplishing highly efficient adenoviral-mediated gene transfer in lymphoma cells [6]. Using this protocol, expression of the wild-type p53 tumor-suppressing gene in lymphoma cell lines with mutant p53 showed increased sensitivity to cytotoxic drug and immuno-mediated toxicity [26]. In the current study, we observed low expression levels of cell surface integrins avβ3 and avβ5 on all lymphoma cells studied, which suggests that the adenoviral entry into these cells may be mediated by CAR, expressed at high levels on Raji and Daudi cells. As a consequence, Raji and Daudi lymphoma cell lines could be transduced with higher efficiency, whereas primary lymphoma cells and normal lymphocytes with low-level expression of CAR were refractory. Turturro et al. have also shown that anaplastic large cell lymphoma cells express high levels of CAR and integrins, which could be transduced by adenoviral vectors with high efficiency [27]. These results indicate the importance of determining the expression levels of CAR and integrins in tissues or cells derived from patients for the generation of adenoviral vector-modified lymhoma cell vaccines. Previously reported transduction efficiencies of adenoviral vector-transduced lymphoma cells were obtained with non-purified viruses [6]. Since this protocol is not feasible for clinical application, the present studies were performed with CsCl-purified viruses and lower transduction efficiencies were achieved. The exact reason for this difference is currently unknown and will be elucidated in the future.

In our hands, human Burkitt's lymphoma cell lines were most efficiently transduced with adenoviral vectors. Expression of IL-2 and IL-12 cytokines in Raji cells transduced at a relatively low MOI of 200 was transient, peaked between 1 and 3 days post-infection, and was detectable up to 10 days. The produced cytokines were assayed for their biological ability to stimulate PBMC from healthy donors in comparison with recombinant cytokines as controls. Our data indicates that adenoviral expressed cytokines were equally effective compared with recombinant cytokines in enhancing the proliferation rates of PBMC. This effect could be blocked by the addition of neutralizing antibodies against either cytokine. In a cytotoxicity assay, IL-2 stimulated PBMC were able to lyse unmodified Raji cells, while IL-12 or the combined IL-2 and IL-12 stimulated PBMC were clearly less effective.

Previously, we have shown that cytotoxic CD8+ NKT cells are readily expandable in vitro in large quantities suitable for adoptive immunotherapy. These activated effector cells have significant cytotoxic activity against human lymphoma xenografts with limited toxicity [8,28]. We have also demonstrated that CD8+ NKT cells can be generated in vitro using either IL-2 or IL-12 [29]. Interestingly, adoptive T cell therapy combined with intratumoral administration of adenoviral expressed IL-12 was shown to have strong synergistic effects against large transplanted tumors [30]. Therefore, expression of IL-2 and IL-12 in lymphoma cells may be used to further increase their sensitivity towards adoptively transferred CD8+ NKT cells in the future.

## Conclusion

This study demonstrates that the generation of recombinant adenovirus modified lymphoma cell vaccines based on lymphoma cell lines expressing IL-2 and IL-12 cytokine genes is technically feasible, induces increases in proliferation rates and cytotoxic activity of co-cultured PBMC, and warrants further development for the treatment of lymphoma patients in the future.

## Competing interests

The author(s) declare that they have no competing interests.

## Authors contributions

OE and DW designed the experiments and performed the experimental studies presented in this paper. PB developed the experimental protocols and assisted in the analysis of the results. CZ, DF, and ISW participated in the design of the study and its coordination. All authors have read and approved this manuscript.
